# Atlanta Academy of Medicine

**Published:** 1879-05

**Authors:** 


### ATLANTA ACADEMY OF MEDICINE.

Atlanta, March 31st, 1879.

Academy met, President J. S. Todd, M.D., in the chair.

Dr. Calhoun presented some further remarks, showing progress of a case of cysticercus, previously reported to the Academy. As then reported, the cysticercus lay between the choroid and retina, and just to the side of the optic nerve, upon which its head rested. A few days ago, however, after wide dilatation of pupil with atropine, he found the relative position of parts the same, though the cysticercus was now twice as long as it had been six months ago; still it lay between the aforementioned coats, and had not protruded into vitreous humor—the retina was pushed out, presenting the characteristic grayish-blue appearance, through which could be seen the entozoon with its short neck and round head, upon which the antennæ or suckers could be seen. Upon any movement of the animal the superjacent retina underwent a distinctly tremulous motion. On account of the already very defective vision, and the ill success attending operations of this nature, together with the patient's unwillingness to undergo any operation, nothing had been done as yet. In reply to the question of the President, Dr. Calhoun stated that the operation consisted simply in opening sclerotic and letting out the entozoon. In the present case, the animal lying to the outer or temporal side of the nerve, he would divide the external rectus muscle completely, and inverting the eye strongly, incise sclerotic and choroid over the animal, and allow the fluid and entozoon to flow out. Great care should be taken not to incise the retina, but the loss of five or six drops of vitrous would be of no moment. Being asked by Dr. J. T. Johnson the size of the cysticercus, Dr. Calhoun said, seen through the refracting media it appeared to be about half an inch long and one-third broad; was not prepared to give its exact dimensions when unmagnified, but promised to compute them for the information of members at some future time.

The President asked Dr. Logan if he had met with any cases of dysentery recently, and what treatment he adopted.

Dr. Logan has not seen any case for some time; should he do so, his main reliance would be upon opium, after evacuating the bowels.

Dr. Calhoun called attention to the use of nitrate of silver injections in dysentery and chronic diarrhœa.

Dr. J. T. Johnson has used this treatment of strong solution locally to rectum in chronic dysentery for the last five years.

Dr. Mack spoke highly of ipecac in ten or fifteen grain doses in acute dysentery, being cautioned from the use of water before and after taking.

Dr. Logan is so thoroughly satisfied with opium that he does not need any other remedy. At the same time, he does not doubt the efficacy of both nitrate of silver and ipecac. Remarked to Dr. Mack that Dr. Woodhull, U. S. A., read his well-known essay on the use of ipecac in dysentery for the first time before this Academy.

In reply to a question, Dr. Logan said he was accustomed to lance gums of children only where they are hot and dry, and the tooth about to come out; until then he can see no possible advantage to arise—besides, unnecessary cutting interferes with the perfect formation of the tooth.

The President remarked that the profession were widely at variance in their views on this subject; he wished to know the experience of members in the use of condensed milk as food for infants.

Dr. Johnson said some children thrive on it, whilst cow's milk disagrees with them; again, some were unable to take the condensed milk, hence he is governed according to test in a particular case.

Dr. Logan uses cow's milk in preference, and it seems to him that children nourished on condensed milk are weak and flabby. As a rule he prefers the milk last drawn from the cow, as containing more oily and less cheesy matter.

Dr. Johnson thinks the milk of the Jersey cow preferable to any other in the latter respect.

Dr. Wilson thinks this is applicable to grown-up children as well, but it would be too rich if not well diluted.

Academy adjourned.

---

Atlanta, April 7th, 1879.

The Academy called to order by the President, Dr. J. S. Todd.

After regular order of business, report of cases called up.

Under this head, Dr. Salm stated that, at the last meeting, there had been some disagreement among the members as to the relative amount of caseine in human and cows milk. Some of the other gentlemen had taken issue with him, and he here appends these tables and accompanying remarks.

Proximate organic principles:

| | |
|---|---|
| caseine, | pepsine, |
| albumen, | sug. of milk. |

Caseine has many properties in common with albumen and fibrine; it exists in greatest abundance in milk and is the basis of cheese; its occurrence in other fluids has not been positively detected; it may be obtained by allowing milk to remain at rest till it is coagulated; it is very perfectly coagulated by rennet. The coagulating power is not due to the acid of the stomach, but to the pepsine resident in it; not coagulated by heat, but is readily precipitated by an acid, contains sulphur but no phosphorus.

Liebig says condensed milk represents a fine article of cows milk with somewhat larger proportion of sugar.

AFTER ANALYSIS OF THE INDUSTRIAL SCHOOLS OF ZURICH.

| Woman's milk contains | | Cow's milk contains |
|---|---|---|
| 68.0 | 48 less | 720.0 water |
| 80 0 | 20 more | 100.0 casiene |
| 60 0 | 10 " | 70 0 salt |
| 80.0 | 10 " | 90.0 sugar of milk |
| 12.0 | 8 " | 20 0 ashes |
| | 48 | |

Herrmann gives in 1000 parts

| | | |
|---|---|---|
| 889.1 | 32 less | 357.0 water |
| 39.2 | 9 more | 48.3 casiene |
| 26.7 | 16 " | (5.8) albumen |
| 43.6 | 3 less | 43 1 fatt |
| 1.4 | 4 more | 40.4 sugar milk |
| | | 5.5 ashes |

COPENHR.

| | | |
|---|---|---|
| 383.6 | 22 less | 361.0 water |
| 25 3 | 13 " | 38.0 butter |
| 34.3 | 34 " | 68.0 casiene |
| 48.2 | 21 " | 29.0 sugar |
| 2.3 | 4 more | 6.1 salt |

ATLANTA, April 14th, 1879.

The Academy was called to order by the President, Dr. J. S. Todd. After dispatching business, Academy proceeded to report of cases:

Dr. John G. Westmoreland reported the case of a woman, forty-six years of age, who has had three children. Is now in third month of pregnancy, and has been troubled for a month or six weeks with what she called a tumor at the vulva. There has been no pain, but the fullness is troublesome, and there has been some hemorrhages, one amounting to half pint. A physician had examined it soon after it appeared, but gave no diagnosis. When Dr. W. examined her lying in the horizontal position, he discovered no protrusion, but by digital examination found that it was a prolapsed vagina. The blood vessels of the vagina, front side, and of the right labium, were very much enlarged. The vagina, from digital examination, was found to be joined on the front and right lateral side, immdiately to the os and not to the side of the cervix. The posterior and left surface was joined naturally. The vagina had a baggy feel in its right and anterior wall.

The tumor of which she complained was caused by this varicose condition of veins, and was formed more on right side than on left, the labium participating in it.

No cutting operation was to be thought of, and so he decided to use some support which would at the same time exert pressure. He decided to use cotton wadding, medicated with eight grains each of tannin and carbolic acid, to an ounce of glycerine, and tamponing with this material. Has been using it for four or five days with benefit, and patient feels much better to-day; so well in fact that she will go out of the city to-morrow. There was no urinary trouble; had been very healthy; a peculiarity with her is that she quickens at three and half months in each pregnancy.

Dr. Todd was called to see a lady in the ninth month of pregnancy, who has a tumor on the right labium, which has recurred about the eighth month in her last three confinements. These do not bleed, however, and grow much smaller at night. He made no examination as he was called for another disorder. She accounted for it by child lying on right side. Dr. Taliaferro inferred that the lady in Dr. Westmoreland's case, suffered from her present trouble as a result of subinvolution from previous pregnancies. Very frequently subinvolution occurs in vagina as well as in uterus. The uterus dropping down in the beginning of second month, its increased weight, probably caused the condition by pressing on the parts and interferring with the circulation. Thinks it probable that the condition will pass away when the uterus rises out of the pelvis; also thinks Dr. Westmoreland right in treatment; attaches some importance to position of women at time of tamponing. She should be in the knee-elbow position, and should be tamponed lightly; has often tamponed and treated locally with mild remedies, lesions of the uterus and vaginia which would have otherwise produced abortion.

Dr. Connally asked Dr. Taliaferro if he had noticed these thrombi occur oftener on the right side?

Dr. Taliafero said he had not.

Dr. Connally related a case similar to that of Dr. Todd, where tumor occurred in right side; large and painful at noon; almost disappeared by morning; had seen another on right side; woman in ninth month.

Dr. Todd would like to know the treatment used by the different members for amenorrhœa, and whether the measures employed to prevent conception would cause the disorders?

Dr. Westmoreland would look into the condition of the organ and into that of the general system, and treat case accordingly. Thinks that practices for prevention of conception would cause amenorrhœa by disturbance of functions of uterus.

Dr. Taliaferro thinks that any means which successfully combat conception cause trouble; has no faith in constitutional treatment in amenorrhœa. In amenorrhœa trouble may be produced by blood or nervous functions, or by derangements of general system. Superinvolution may produce amenorrhœa and the remedies are, the introduction of probe, tent or galvanic stem, composed of copper on one side and zinc on other, as devised by Sir. J. Y. Simpson and modified by himself. In the event it is caused by deficient blood and nerve supply, the galvanic stem increases it by stimulation. Has now the case of a woman who is insane, and when he began treating her some months ago, the uterus was only two inches long. He applied the galvanic stem which she has worn since, and the uterus has increased to two and a half inches in length. Menstruation, which had ceased for a long time, previously to commencing treatment, now occurs occasionally, but not regularly, and the mind has improved. He hopes when menstruation becomes regular the mind will be entirely restored; but is not too hopeful, as there is hereditary insanity existing in her family.

In another case of insanity, amenorrhœa with a uterus of only one and three-quarter inches existed; when he first saw her there was a slight abrasion of os; this was successfully treated with iodine; the galvanic stem was then introduced, and worn for three months; improvement dated from time of using the stem; after first menstruation, mind became somewhat better, and in a few more menstruations the mind was entirely restored. When treatment began she was extremely debilitated, but is now a strong, healthy woman, following her avocation of music teacher.

Dr. Todd related a case of a woman, married ten years; had two children; last one six years of age; after birth of last child, she began to employ means to prevent conception; after four years of these practices, menstruation grew less and less, until now it is extremely scanty; does not know what means she employed; suffers greatly from headache and nervous disorders. In an article in Atlanta Medical Journal last year, some New York physician had maintained that the seminal fluid was necessary to healthful cohabitation. That it acted as a sedative to the female parts.

Dr. Taliaferro is satisfied that in habitual onanism, the woman does not have her orgasm, and suffers in consequence various nervous disorders.

Dr. Salm asks opinion of gentlemen in case of epilepsy. The patient first had an attack when picking cotton in the hot sun; her menstruations have been very profuse and protracted since; her flow is excessive for five days, and for three days longer it amounts to as much as that of an ordinary menstruation; thinks epilepsy might be caused by the dysmenorrhœa; attacks come on monthly, and last during the time her menses are on; she does not foam at her mouth, but becomes unconscious; he has not seen her in an attack; Dr. Taliaferro thinks that they are hysterical in their nature; thinks that patient sometimes becomes unconscious in hysterical convulsions; hysteria simulates all forms of epilepsy; comes on at menstrual periods or are aggravated at that time; may finally occur same at all times; in hysterical attacks they usually laugh just before or just after the attack.

Dr. P. asked if dysmenorrhœa could cause epilepsy?

Dr. Taliaferro thought not.

Academy adjourned.

